# Psychological and Behavioral Responses in South Korea During the Early Stages of Coronavirus Disease 2019 (COVID-19)

**DOI:** 10.3390/ijerph17092977

**Published:** 2020-04-25

**Authors:** Minjung Lee, Myoungsoon You

**Affiliations:** 1 Department of Public Health Sciences, Graduate School of Public Health, Seoul National University, Seoul 08826, Korea; leciel84@snu.ac.kr; 2 Institute of Health and Environment, Seoul National University, Seoul 08826, Korea

**Keywords:** pandemics, coronavirus, public health emergency preparedness, perceived risk, efficacy belief, precautionary behaviors

## Abstract

*Background:* The psychological and behavioral responses during the early stage of Coronavirus disease 2019 (COVID-19) in South Korea were investigated to guide the public as full and active participants of public health emergency preparedness (PHEP), which is essential to improving resilience and reducing the population’s fundamental vulnerability. *Methods:* Data were collected through an online survey four weeks after the Korea Centers for Disease Control and Prevention (KCDC) confirmed the first case in South Korea; 973 subjects were included in the analysis. *Results:* Respondents’ perceived risk of COVID-19 infection; the majority of respondents reported that their perceived chance of infection was “neither high nor low” (51.3%). The average perceived severity score was higher than perceived susceptibility; 48.6 % reported that the severity would be “high,” while 19.9% reported “very high.” Many respondents reported taking precautions, 67.8% reported always practicing hand hygiene, and 63.2% reported always wearing a facial mask when outside. Approximately 50% reported postponing or canceling social events, and 41.5% were avoiding crowded places. Practicing precautionary behaviors associated strongly with perceived risk and response efficacy of the behavior. *Conclusions:* Our study confirmed the significance of the psychological responses, which associated with behavioral responses and significantly influenced the public’s level of public health emergency preparedness regarding the COVID-19 pandemic. This result has consequences not only for implementing public health strategies for the pandemic but also for understanding future emerging infectious diseases.

## 1. Introduction

Starting in December 2019, an increasing number of cases of a novel coronavirus (COVID-19) were identified in Wuhan, a large city of 11 million people located in central China [1,2]. The COVID-19 epidemic was not limited to China, however. On 20 January, South Korea confirmed its first case [3], and by 30 January, the World Health Organization (WHO) had declared the outbreak of the COVID-19 to be a global health emergency. The WHO acknowledged the virus’ risk to countries beyond China and identified the need for a better-coordinated international response to the outbreak [4,5]. As of 15 April, the COVID-19 epidemic continues to spread globally, with more than 2,100,000 confirmed cases in 106 countries, including Japan, Italy, Spain and the USA.

Considering that the definition of a public health emergency is one that’s “scale, timing, or unpredictability threatens to overwhelm routine capabilities” [6], COVID-19 outbreak in Korea can reasonably be classified as a public health emergency; the virus can threaten and overwhelm a population’s capabilities in terms of scale and unpredictability. In cases of public health emergencies, implementing public health emergency preparedness is critical. Public Health Emergency Preparedness (PHEP) refers to the level of readiness for public health and health management systems, communities, and individuals to prevent, respond to, and recover from public health emergencies [6]. PHEP constitutes a broad range of prevention, mitigation, and recovery activities and emphasizes that such goals cannot be accomplished by the government alone, but must also involve the public [6]. Therefore, engaging the public is one of the most critical elements of PHEP and manifests by heightened risk perceptions [7,8], increased knowledge and awareness about specific threats [7,9], and the implementation of precautionary measures [9,10,11]. Guiding the public to become full and active participants in PHEP is essential to improving resilience and reducing the population’s overall vulnerability [6]. As information about the COVID-19 virus (transmission dynamics, incubation time, epidemic doubling time, and basic reproductive frequency) remains scarce and uncertain [1], and there are no antiviral remedies or vaccines with proven efficacy, the public’s involvement in PHEP plays a critical role in the prevention of the spread of the epidemic.

Recognizing the significance of PHEP, we investigate the psychological and behavioral responses of the public related to several aspects of PHEP during the COVID-19 outbreak. The public’s response during a pandemic provides useful information for health risk communication and achieving successful changes in public behavior [12]. Several published studies have addressed psychological and behavioral responses to pandemic diseases [13,14,15]. Most of the research related to the COVID-19 outbreak focuses on epidemiology [1], clinical characteristics of infected patients [2,3], and mental health of the Chinese population [16,17,18]. To our knowledge, this paper is the first to report on how South Korea’s population has responded to the COVID-19 outbreak and implemented control measures in the relatively early stage of the outbreak.

For psychological responses, we focus on perceived risk, which has two components, perceived susceptibility and severity. Perceived risk refers to an individual’s belief of vulnerability to a particular risk [19]. Perceived susceptibility refers to beliefs about the likelihood of experiencing an illness, whereas severity refers to beliefs about the seriousness or magnitude of an illness [20,21]. The greater the risk an individual perceives, the more motivated he or she should be to engage in protective behaviors. We also investigate efficacy belief, which refers to the perceived benefits from engaging in a particular behavior [20,21], and what motivates one to adopt self-protection behavior. 

Perceived risk and efficacy belief both have the potential to determine individuals’ behavioral response, such as practicing preventive behaviors. Health behavior models, such as Protection Motivation Theory (PMT) [22] and Health Belief Model (HBM) [23], propose that risk perception, together with other concepts such as perceived benefits and barriers, are key contributors to people’s willingness to make behavioral changes. Derived from the extended parallel process model (EPPM) [21], Risk Perception Attitude (RPA) framework [20,24] hypothesizes that an individual’s efficacy beliefs act as a key factor along with perceived risk in driving behavioral changes. Conceptually, efficacy beliefs comprise (a) response efficacy or outcome expectations, which are the beliefs about the effectiveness of the recommended response in deterring, impeding, or averting a health risk, and (b) self-efficacy, which is an individuals’ perceived ability to exert personal control [21]. Multiple studies have tested the role of perceived risk and efficacy belief on health behaviors related to prevention of HIV, HPV [25], cancer [26], diabetes [27,28], preventive behaviors related to nutrition [26], maternal physical activity [29], safe driving [30] and adopting safe behaviors during use of chemical products [31]. 

For behavioral response, we focus on practicing precautionary behaviors among the general public. Non-pharmaceutical public health interventions, such as hand hygiene and wearing masks, are performed to inhibit human-to-human transmission [32,33]. Following proper handwashing hygiene is an activity that is often recommended by health authorities to prevent transmission of the virus during infectious disease outbreaks [34], most frequently researched preventive behaviors [35], and have proven to affect the spread of pandemics [36] greatly. Moreover, the protective effect of wearing facial masks to reduce respiratory virus transmission is widely supported in the literature [37,38]. Practicing hand hygiene and wearing facial masks were recommended by the Korean government.

Avoidance behaviors, such as canceling or postponing social events, reducing the use of public transport, keeping children out of school, and avoiding crowded places due to fear of transmission frequently occur during pandemic outbreaks [34,39,40]. Individual avoidance behaviors that limit contact with others are forms of social distancing known as ‘informal social distancing’ [41]. Previous studies suggest that social distancing behaviors among large numbers of people within a population can damage daily lives, lead to adverse social implications, and even lead to greater risk [40,42]. Therefore, special attention should also be paid to social distancing from the PHEP perspective during a pandemic. Regarding the behavioral response, we focus on practicing recommended behaviors (i.e., practicing hand hygiene and wearing facial masks), as well as social distancing (i.e., reducing the use of public transport, avoiding crowded places, and postponing or canceling social events). 

The present study investigates the psychological and behavioral responses to the COVID-19 outbreak among South Korea’s general population within the first four weeks of the COVID-19 outbreak. We examine the perceived risk, efficacy belief about precautionary behaviors, and the execution level of precautionary behaviors, and identify sociodemographic and psychological factors that contribute to behavioral response. Our goal is to improve the collective understanding of public response during the early stage of the pandemic, COVID-19. The results of the present study will deepen our understanding of the psychological and behavioral response of the public in the view of PHEP.

## 2. Methods

### 2.1. Data Collection

We adopted a cross-sectional survey design to evaluate the public’s psychological and behavioral responses during the COVID-19 epidemic using an anonymous online questionnaire. The survey was conducted via an online platform from a research company called Korea Research. Korean residents ages 18 years and older were recruited. Potential respondents were invited via e-mail and text messages. Proportionate quota sampling was used to characterize age, gender, and population region. A total of 973 subjects completed the surveys and were included in the analysis. The survey and consent to participate were approved by the National Medical Center Institutional Review Board (IRB No. H-2002-111-003). The data collection took place over three days (25–28 February), four weeks after the Korea Centers for Disease Control and Prevention (KCDC) confirmed the first case at the early stage of the epidemic. 

### 2.2. Questionnaire

Previous surveys on the psychological and behavioral responses of emerging infectious diseases were reviewed [7,8,9,10,13,14], and the authors included additional questions related to COVID-19. The structured questionnaire consisted of questions that covered several areas: (1) perceived risk related to COVID-19; (2) efficacy belief on precautionary measures; (3) COVID-19 health risk communication; (4) precautionary behaviors practiced against COVID-19 in the past seven days; and (5) socio-demographic data.

Socio-demographic characteristics of respondents included gender (1 = male, 2 = female), age, education level (values ranged from 1 (High school or below) and 2 (Some college and above)) and monthly household income (values ranged from 1 (200 Korean thousand won or below) to 4 (600 Korean thousand won or above). We collected information about the respondents’ residence (City = 1, Town = 2), the presence of children younger than elementary school in the home (yes = 1, none = 0), and subjective health status (Poor = 1, Moderate = 2, Good = 3) were also investigated. We also investigated respondents’ perceived social support regarding whether they would likely have support if they were isolated due to COVID-19 (yes = 1, no = 0) (Table 1).

Following Rimal and Juon (2010), perceived risk of COVID-19 infection comprised both perceived susceptibility, which signifies individuals’ beliefs about their possibility to infection, and perceived severity, which signifies the seriousness of infection [24]. Respondents were asked, “What do you think is the possibility of a COVID-19 infection?” and, “What do you think is the severity if COVID-19 infects you?” Responses were rated on a 5-point Likert-type scale ranging from 1 to 5 with 1 = not at all and 5 = extremely) (Table 2).

Precautionary behaviors to the threat of the COVID-19 fell into one of the two following categories: (1) preventive measures (e.g., wearing facial masks, practicing hand hygiene); or (2) social distancing behaviors (e.g., reducing the use of public transport, avoiding crowded places and postponing or canceling social events). For efficacy belief, respondents answered, “To what extent do you think each precautionary behavior is an effective way to reduce the risk of COVID-19 infection?” and responses were rated on a 5-point Likert-type scale ranging from 1 to 4 with 1 = not at all and 4 = extremely) (Table 2). 

COVID-19 related health risk communication was examined using two variables adapted from Clarke and McComas [43], information sufficiency, and perceived information gathering capacity [43,44]. Perceived information sufficiency is a perceived knowledge deficit or a gap between what one currently knows and what one should know. Respondents rated the following question on a 5-point Likert scale, “At this time, all the information I have about COVID-19 meets my needs” (1 = not at all, 5 = very much). Perceived information gathering capacity is a person’s perceived ability to locate and process information about an issue if desired [44]. Respondents rated the following question on a 5-point Likert scale: “Was it easy or hard to find information about COVID-19 when you need it?” (1 = very hard, 5 = very easy) (Table 2).

To assess precautionary behaviors practiced against COVID-19, we measured the self-reported practice of the study participants using five questions to assess how frequently they engaged in those behaviors. Specifically, we were interested in participants’ use of the following: (1) using preventive measures (e.g., wearing facial masks, practicing hand hygiene); and (2) social distancing behaviors (e.g., reducing the use of public transport, avoiding crowded places, and postponing or canceling social events) during the previous week using a 4-point Likert-type scale (never, sometimes, often, and always) (Table 2). 

### 2.3. Statistical Analysis

We conducted statistical analyses using R version 3.5.1 (R Foundation for Statistical Computing, Vienna, Austria). All results of quantitative variables were reported either as mean (*M*), standard deviation (*SD*) or frequency (percentage%). Multivariate linear regression analysis to examine the effect of sociodemographic factors and COVID-19 related health risk communication factors on perceived risk (perceived susceptibility and severity) was performed. Additionally, we used a multivariate linear regression model to estimate the associations among the noted sociodemographic factors (i.e., gender, age, educational level, monthly household income, residence, and presence of young children), subjective health status, social support, perceived risk, and response efficacy toward each precautionary behavior practiced against COVID-19.

## 3. Results

### 3.1. Descriptive statistics

Among the 973 respondents, there were 486 men (49.9%) and 487 women (50.1%), with a mean age of 46.41 years (*M* = 46.41, *SD* = 14.94) (Table 1). A majority of the respondents had only a high school education (53.4%), followed by those with at least some college education (46.6%). The most common monthly household income was approximately 2.00–3.99 million won ($1688–$3369 USD; 34.1%), followed by 4.00–5.99 million won ($3377–$5057 USD; 27.4%) and over 6.00 million won ($5065 USD; 26.7%), Table 1). Among the respondents, 85.1% lived in a city, and about 10.7% had young children in the home. Most respondents reported their health status as being subjectively good (53.3%) or moderate (40.2%). 72.0% of the respondents reported they could get someone else’s support if they were isolated due to COVID-19, but 28.0% said they would not have social support.

### 3.2. Risk Perception Related to COVID-19

Respondents perceived the risk of becoming infected with COVID-19 (perceived susceptibility) as being higher than “low” (score = 2) (*M* = 2.87, *SD* = 0.88). Only 3.6% reported a perceived chance of infection is “very high” (score = 5) and 16.0% reported “high” (score = 4). The majority of respondents reported that the chance of infection is “neither high nor low” (51.3%). The average perceived severity score was higher than perceived susceptibility, which was close to “high” (score = 4) (*M* = 3.79, *SD* = 0.87). However, among the participants, 48.6% reported that the severity would be “high” (score = 4), and 19.9% reported “very high” (score = 5).

### 3.3. Response Efficacy to Precautionary Behaviors

Among the five precautionary behaviors, perceived benefits from engaging in hand hygiene were the highest (*M* = 3.80, *SD* = 0.42), followed by wearing facial masks (*M* = 3.72, *SD* = 0.49). However, the response efficacy on social distancing, such as reducing the use of public transport, was the lowest (*M* = 3.55, *SD* = 0.62), followed by avoiding crowded places (*M* = 3.66, *SD* = 0.56) among the precautionary behaviors.

### 3.4. Precautionary Behavior

The most frequently practiced precautionary behavior was hand hygiene, such as washing hands frequently and using hand sanitizers, 67.8% reported they always practiced. 63.2% reported always wearing a facial mask when outside. Respondents reported that hand hygiene was their most precautionary behavior, followed by wearing facial masks when outside. For social distancing, postponing ore canceling social events was the most practiced behavior (50.2% reported “always”), followed by always avoiding crowded places (41.5%). However, only 38.7% reported they always reduced using public transportation.

### 3.5. COVID-19 Related Health Risk Communication

Regarding participants perceived sufficiency of health information related to COVID-19 (*M* = 3.10, *SD* = 1.02), only 5.0% reported they were able to get information as much as they needed. 21.5% of the respondents reported that information sufficiency was low (score = 2), and 7.0% reported it was very low (score = 1). For perceived information gathering capacity (*M* = 3.55, *SD* = 0.87), the majority of respondents found it “quite easy” to find needed COVID-19 information (46.7%), followed by “neither easy nor hard” (32.4%). However, 10.4% of the respondents reported it was hard or very hard to find COVID-19 information when needed.

### 3.6. Influencing Factors on Risk Perception Related to COVID-19

Influencing factors on risk perception (i.e., perceived susceptibility and perceived severity) among sociodemographic groups and health risk communication variables were assessed (Table 3). Sociodemographic variables and COVID-19 related health risk communication variables accounted for approximately 7% of the variance in perceived susceptibility on COVID-19, F (10, 962) = 8.48, adjusted R^2^ = 0.07, *p* < 0.001. As shown in Table 3, age (β = −0.01, *p* < 0.01), subjective health (β = −0.15, *p* < 0.001), social support (β = −0.13, *p* < 0.05), perceived information sufficiency (β = −0.11, *p* < 0.001) and information gathering capacity (β = −0.09, *p* < 0.05) were negative and significant individual predictors of perceived susceptibility to COVID-19. Gender, Education level, monthly household income, residence and presence of children did not influence perceived susceptibility. Similar to perceived susceptibility, age (β = −0.01, *p* < 0.01), subjective health (β = −0.15, *p* < 0.001), social support (β = −0.13, *p* < 0.05), perceived information sufficiency (β = −0.11, *p* < 0.001) and perceived information gathering capacity (β = −0.09, *p* < 0.05) were negative and significant individual predictors of perceived severity to COVID-19. Sociodemographic factors and health risk communication variables accounted for approximately 11% of the variance in perceived severity on COVID-19, F (10, 962) = 13.82, adjusted R^2^ = 0.11, *p* < 0.001. Among the influencing factors, subjective health followed by social support showed the greatest significant impact. 

### 3.7. Influencing Factors on Practicing Precautionary Behaviors Related to COVID-19

We used multivariate regression to test the association between practicing precautionary behaviors and respondents’ sociodemographic factors, subjective health, social support, perceived susceptibility and severity, and responsive efficacy of each behavior (Table 4). Regarding precautionary behaviors recommended by public health, gender (β = 0.26, *p* < 0.001), education level (β = 0.19, *p* < 0.001), perceived severity (β = 0.07, *p* < 0.05), and response efficacy (β = 0.41, *p* < 0.001) were positive and significant individual predictors of wearing facial masks. The variables accounted for approximately 13% of the variance in adoption of wearing facial masks, F (11, 961) = 14.48, adjusted R^2^ = 0.13, *p* < 0.001. For practicing hand hygiene, gender (β = 0.19, *p* < 0.001), age (β = −0.01, *p* < 0.001), monthly household income (β = 0.02, *p* < 0.05), perceived severity (β = 0.08, *p* < 0.001) and response efficacy (β = 0.35, *p* < 0.001) were positively associated to the practice. The variables accounted for approximately 12% of the variance in practicing hand hygiene, F (11, 961) = 12.61, adjusted R^2^ = 0.12, *p* < 0.001.

Men and respondents with low socio-economic status are vulnerable in both recommended preventive measures. Among psychological factors, having higher perceived severity and response efficacy increased respondents’ frequency of performing the recommended behaviors.

Factors affecting the frequent practices of social distancing behaviors (e.g., reducing the use of public transport, avoiding crowded places and postponing or canceling social events) have shown different patterns from recommended precautions (Table 5). Regarding the avoidance of public transportation use, only the respondent’s residence (town) (β = 0.22, *p* < 0.05) and response efficacy were related significantly (β = 0.44, *p* < 0.001). Sociodemographic variables and psychological variables accounted for approximately 6% of the variance in reducing the use of public transport, F (11, 961) = 6.36, adjusted R^2^ = 0.06, *p* < 0.001.

For avoiding crowded places, age (β = −0.01, *p* < 0.001) was negative and significant individual predictor, presence of children (β = 0.32, *p* < 0.01), perceived severity (β = 0.12, *p* < 0.01) and response efficacy (β = 0.48, *p* < 0.001) were positive and significant individual predictors. The variables explained the behavior for approximately 8% of the variance in avoiding crowded places, F (11, 961) = 8.73, adjusted R^2^ = 0.08, *p* < 0.001. Lastly, postponing or canceling social events were influenced positively and significantly by respondent’s gender (β = 0.21, *p* < 0.001), perceived severity (β = 0.12, *p* < 0.001), response efficacy (β = 0.47, *p* < 0.001) and negatively by respondent’s age (β = −0.01, *p* < 0.05). The variables explained the behavior for approximately 10% of the variance in avoiding crowded places, F (11, 961) = 10.57, adjusted R^2^ = 0.10, *p* < 0.001. Of all the variables, only the efficacy belief related significantly to all recommended and social distancing behaviors and had the greatest impact. 

## 4. Discussion

Our survey results provide valuable insights into psychological and behavioral responses related to the COVID-19 pandemic, an emerging infectious disease, in South Korea in 2020. Among the participants of our study, a substantial proportion reported that they perceived the seriousness of COVID-19 infection (perceived severity) as high; however, the respondents’ beliefs about their possibility to infection (perceived susceptibility) were relatively low. The results of this study suggest that many people take precautions, regardless of whether public health authorities recommend them to do so, to reduce the risk of COVID-19 infection. The results also identified sociodemographic factors that influence perceived risk and psychological factors related to the adoption of precautionary behaviors.

There are several interesting findings worth noting. First, the psychological response of the public regarding perceived risk (susceptibility and severity) is not directly proportional to the hazardous nature of the virus. A study of early assessment of the COVID-19 transmissibility reported the current COVID-19 epidemic has a substantial potential for causing a pandemic [45] and appears that the mortality of the virus was 11% [46], which is low compared to MERS-CoV, which has been reported to exceed more than 35% [47].

COVID-19 has a high potential for transmission and relatively low mortality; however, the public’s perceived susceptibility was low and perceived severity was high. The psychological responses are in agreement with those reported in similar studies on COVID-19. A recent survey report of the public in Hong Kong [48] reported that about 89% of the respondents regarded themselves as likely to become infected with COVID-19, and even more respondents (97%) considered the symptoms of COVID-19 infection to be serious. Interestingly, the results of a survey of the Chinese population of two cites of Wuhan and Shanghai revealed that the Shanghai respondents reported significantly lower perceived susceptibility and higher perceived severity than their counterparts in Wuhan [49]. Moreover, the present study examined sociodemographic and health risk communication variables related to perceived risk. Information sufficiency and perceived information gathering capacity were associated to the level of perceived susceptibility and severity. As a result, this study confirms that health authorities must make efforts to provide enough information to help the public form an appropriate level of risk perception in terms of perceived susceptibility and perceived severity. Moreover, additional support for vulnerable populations should be made by providing targeted, tailored messages with the appropriate literacy.

Second, our results show that psychological responses are essential for behavioral responses and can significantly influence the level of public preparedness in a public health emergency such as the COVID-19 pandemic. Psychological responses, perceived risk and efficacy belief influenced the adoption of precautionary behavior. Therefore, not only behavioral response but psychological responses of the general population play an important role in the control of the outbreak. Previous studies have confirmed that not everyone has the same response to health risks, and risk perceptions alone cannot explain health behaviors, leading to the hypothesis that an individual’s efficacy beliefs influence his or her health behaviors [20]. In the case of public health emergencies, a literature review of studies assessing the factors that influence preventive behavior during pandemic situations highlighted that perceived risk of the disease as well as believing in the effectiveness of protective measures are the main factors influencing the adoption of the protective measures [50,51]. The present study provides an additional contextual link between psychological and behavioral responses from a PHEP perspective. The results of this study suggest that emphasizing risk perception and efficacy beliefs in the COVID-19 prevention message can motivate public to engage in preventive measures.

Drake, Chew, and Ma suggested the concept of “social learning,” which refers to the collective effects of various processes including the dissemination of information to the public, aggressive public health education, and the implementation of public health policies that determine the rate of infectious populations [52]. Thus, communicating with the public and inviting them as an important player in PHEP is critically important in the early stages of a pandemic. Social learning highlights the effectiveness of timely public education, thereby leading to early participation by the public. The sooner the public becomes involved, the sooner the pandemic can end. Therefore, this study has strength in providing information in preparing risk communication messages and strategies in the early stage of pandemic. Risk communication strategies to increase the belief in the efficacy of practicing preventive actions and perceived risks of the public are essential.

Finally, we found that the behavioral response was high and that respondents reported practicing preventive measures and social distancing. Among the tested precautionary behaviors, the Korea Centers for Disease Control and Prevention (KCDC) strongly recommended wearing facial masks when leaving one’s home and practicing hand hygiene. These measures aim to maximize prevention and minimize the occurrence of new infections, which helps to protect the healthy population against viral infection. Considering that the survey was conducted before social distancing was first proposed (28 February), many individuals have since tried to reduce their risk of acquiring COVID-19.

The high behavioral response might relate to the South Koreans’ experience with the Middle East Respiratory Syndrome (MERS) in 2015, as the COVID-19 outbreak brings back memories of the MERS. Between the first documented occurrence of MERS infection (20 May 2015) and when the last case was diagnosed (4 July 2015), there were 186 confirmed cases, with 38 deaths and 16,752 people quarantined [53]. In situations of high uncertainty, especially in the early stages of an epidemic, people find it difficult to find the information they need if certain levels of government surveillance are lacking [54], the information provided is insufficient, scant, or unreliable [55,56]. This outcome was, unfortunately, the case during the 2015 South Korean MERS outbreak [11]. The 2015 MERS outbreak in South Korea resulted in significant damage to the population, widespread distrust, and societal levels of high stress. Nevertheless, the MERS pandemic has provided many important lessons about PHEP to the public as well as to the public health authorities [11]. 

Our study has several limitations. First, our analyses did not extensively explore psychological factors such as perceived barriers to practicing preventive behaviors, or communication factors like seeking information, using the media, or processing information. Second, we were unable to directly compare the perceived risk of COVID-19 with MERS or any other infectious diseases for the same study participants. Therefore, it is difficult to compare the absolute values of the perceived risk. Third, efficacy belief is conceptualized to include both response efficacy and self-efficacy, and the latter was not explored in this study. Our study aimed to identify psychological factors related to behavioral responses, rather than testing theoretical hypothesis. However, this still remains as a limitation of this study. 

## 5. Conclusions

Based on the results of our survey, we demonstrated that the public’s perception of risk related to COVID-19 infection was high and that practicing preventive measures and social distancing behaviors were frequent. We identified factors that influence perceived risk and the practice of precautionary behaviors; our findings align with studies from other countries and those addressing former infectious diseases [7,13,14,15]. Our study’s findings also confirmed the importance of psychological responses, which associated with behavioral responses and significantly influenced the public’s level of PHEP regarding the COVID-19 pandemic. This study is one of the first conducted in the early stage of the COVID-19 pandemic in South Korea. The results regarding psychological and behavioral responses, as reported by the study participants, have consequences for implementing public health risk communication for the COVID-19 pandemic and for improving the collective understanding of emerging infectious diseases. The results of the present study will deepen our understanding of the psychological and behavioral response of the public in the view of PHEP.

## Figures and Tables

**Table 1:** Descriptive statistics of survey respondents (*n* = 973).

Characteristics	No.	%
Gender	973	
Male	486	49.9
Female	487	50.1
Age groups	*M* = 46.41	*SD* = 14.94
18–29	172	17.7
30–39	166	17.1
40–49	192	19.7
50–59	199	20.5
≥60	244	25.1
Education		
Under high school	520	53.4
College and above	453	46.6
Monthly household income ^a^		
Under 200	114	11.7
200–399	332	34.1
400–599	267	27.4
≥600	260	26.7
Residence		
City	828	85.1
Town	145	14.9
Presence of children		
Children before elementary school	104	10.7
None	869	89.31
Health status		
Bad	63	6.5
Moderate	391	40.2
Good	519	53.3
Social support		
None	272	28.0
More than one person	701	72.0

Notes: ^a^ South Korean 10,000 won (USD1 = KRW 1185.00).

**Table 2:** Psychological and behavioral responses on COVID-19.

Variable	*M*	*SD*
Perceived risk		
Perceived susceptibility	2.87	0.88
Perceived severity	3.79	0.87
Response efficacy of precautionary behavior		
Wearing facial masks	3.72	0.49
Hand hygiene	3.80	0.41
Reducing the use of public transport	3.55	0.62
Keeping away from crowded places	3.66	0.56
Postponing or canceling social events	3.66	0.57
Practicing “always” of precautionary behavior	No.	%
Wearing facial masks	615	63.2
Hand hygiene	660	67.8
Reducing the use of public transport	377	38.7
Keeping away from crowded places	404	41.5
Postponing or canceling social events	488	50.2

**Table 3:** Results of multivariate linear regression analysis of perceived risk for COVID-19.

Variables	Perceived Susceptibility					Perceived Severity				
	B (95% CI)	Std. Error	Beta	t	*p*-Value	B (95% CI)	Std. Error	Beta	t	*p*-Value
Constant	4.10 (3.90–4.81)	0.24		17.43	0.00	4.46 (4.12–5.00)	0.24		18.22	0.00
Gender (Male:1, Female:2)	−0.01 (−0.11–0.11)	0.06	0.00	−0.09	0.93	−0.01 (0.02–0.23)	0.06	0.00	−0.09	0.93
Age (in years)	−0.01 (−0.09–−0.02)	0.00	−0.09	−2.76	0.01	−0.01 (0.05–0.13)	0.00	−0.09	−2.76	0.01
Education level	−0.04 (−0.16–0.06)	0.05	−0.02	−0.84	0.39	−0.05 (−0.12–0.10)	0.06	−0.03	−0.85	0.40
Monthly household income	0.01 (−0.02–0.04)	0.01	0.02	0.74	0.45	0.01 (−0.03–0.02)	0.01	0.02	0.75	0.45
Residence (City:1, Town:2)	−0.01 (−0.17–0.14)	0.08	0.00	−0.14	0.89	−0.01 (−0.18–0.11)	0.08	0.00	−0.14	0.89
Presence of children	0.03 (−0.15–0.21)	0.09	0.01	0.31	0.75	0.03 (−0.12–0.22)	0.09	0.01	0.31	0.75
Subjective health	−0.15 (−0.27–−0.09)	0.04	−0.13	−4.09	0.00	−0.15 (−0.23–−0.05)	0.04	−0.13	−4.09	0.00
Social support	−0.13 (−0.26–−0.01)	0.06	−0.07	−2.05	0.04	−0.13 (−0.32–−0.05)	0.06	−0.07	−2.05	0.04
Information sufficiency	−0.11 (−0.18–−0.04)	0.04	−0.13	−3.16	0.00	−0.11 (−0.25–−0.11)	0.04	−0.13	−3.16	0.00
Perceived information gathering capacity	−0.09 (−0.17–−0.01)	0.04	−0.08	−2.03	0.04	−0.09 (−0.14–0.02)	0.04	−0.08	−2.03	0.04
Adjusted R^2^	0.07					0.11				

**Table 4:** Results of multivariate linear regression analysis of precautionary behavior.

Variables	Wearing Facial Masks					Hand Hygiene				
	B (95% CI)	Std. Error	Beta	t	*p*-Value	B (95% CI)	Std. Error	Beta	t	*p*-Value
Constant	1.07 (0.52–1.58)	0.29		3.74	0.00	1.42 (0.96–1.93)	0.26		5.52	0.00
Gender (Male:1, Female:2)	0.26 (0.17–0.35)	0.05	0.17	5.61	0.00	0.19 (0.12–0.27)	0.04	0.15	4.90	0.00
Age (in years)	0.00 (−0.06–0.01)	0.00	−0.03	−0.99	0.32	−0.01 (−0.08–−0.02)	0.00	−0.12	−3.54	0.00
Education level	0.19 (0.10–0.28)	0.05	0.12	3.99	0.00	0.06 (−0.01–0.14)	0.04	0.05	1.58	0.11
Monthly household income	0.00 (−0.02–0.03)	0.01	0.01	0.27	0.79	0.02 (0.00–0.04)	0.01	0.06	2.03	0.04
Residence (City:1, Town:2)	−0.05 (−0.18–0.07)	0.06	−0.02	−0.82	0.41	0.03 (−0.07–0.14)	0.05	0.02	0.65	0.51
Presence of children	0.10 (−0.05–0.25)	0.07	0.04	1.31	0.19	0.04 (−0.08–0.16)	0.06	0.02	0.72	0.47
Subjective health	0.01 (−0.08–0.07)	0.03	0.01	0.34	0.73	0.04 (−0.02–0.09)	0.03	0.04	1.37	0.17
Social support	0.01 (−0.09–0.11)	0.05	0.01	0.25	0.80	0.01 (−0.08–0.09)	0.04	0.00	0.12	0.91
Perceived Susceptibility	0.04 (−0.01–0.10)	0.03	0.05	1.57	0.12	0.03 (−0.01–0.08)	0.02	0.05	1.41	0.16
Perceived Severity	0.07 (0.02–0.13)	0.03	0.08	2.29	0.02	0.08 (0.03–0.13)	0.02	0.11	3.34	0.00
Response Efficacy	0.41 (0.31–0.50)	0.05	0.27	8.60	0.00	0.35 (0.26–0.45)	0.05	0.23	7.26	0.00
Adjusted R^2^	0.13					0.12				

**Table 5:** Results of multivariate linear regression analysis of social distancing behavior.

Variables	Reducing the Use of Public Transport					Keeping Away from Crowded Places					Postponing or Canceling Social Events				
	B	Std. Error	Beta	t	*p*-Value	B	Std. Error	Beta	t	*p*-Value	B	Std. Error	Beta	t	*p*-Value
Constant	0.03 (−0.61–0.99)	0.45		0.07	0.94	0.24 (−0.48–1.13)	0.45		0.54	0.59	0.46 (−0.15–1.28)	0.39		1.18	0.24
Gender (Male:1, Female:2)	0.01 (−0.13–0.17)	0.08	0.01	0.18	0.86	0.09 (−0.05–0.24)	0.07	0.04	1.24	0.22	0.21 (0.09–0.34)	0.06	0.10	3.21	0.00
Age (in years)	0.00 (−0.05–0.06)	0.00	0.00	0.05	0.96	−0.01 (−0.14–−0.04)	0.00	−0.11	−3.38	0.00	−0.01 (−0.09–0.00)	0.00	−0.07	−2.25	0.02
Education level	0.07 (−0.08–0.23)	0.08	0.03	0.87	0.39	0.12 (−0.02–0.28)	0.08	0.05	1.62	0.11	0.11 (−0.02–0.24)	0.07	0.05	1.64	0.10
Monthly household income	0.01 (−0.03–0.05)	0.02	0.01	0.39	0.70	0.02 (−0.02–0.06)	0.02	0.03	0.89	0.38	0.02 (−0.02–0.05)	0.02	0.03	0.92	0.36
Residence (City:1, Town:2)	0.22 (0.01–0.43)	0.11	0.06	2.02	0.04	0.08 (−0.12–0.28)	0.10	0.02	0.76	0.45	−0.02 (−0.20–0.15)	0.09	−0.01	−0.28	0.78
Presence of children	0.19 (−0.06–0.43)	0.12	0.05	1.50	0.13	0.32 (0.08–0.55)	0.12	0.08	2.69	0.01	0.15 (−0.06–0.35)	0.10	0.04	1.45	0.15
Subjective health	0.08 (−0.03–0.22)	0.05	0.05	1.48	0.14	0.09 (−0.02–0.23)	0.05	0.06	1.84	0.07	0.02 (−0.06–0.15)	0.04	0.02	0.49	0.62
Social support	−0.05 (−0.22–0.12)	0.09	−0.02	−0.58	0.56	−0.07 (−0.22–0.11)	0.08	−0.02	−0.78	0.44	−0.01 (−0.15–0.14)	0.07	0.00	−0.10	0.92
Perceived Susceptibility	0.06 (−0.04–0.15)	0.05	0.04	1.25	0.21	0.00 (−0.10–0.08)	0.05	0.00	−0.10	0.92	−0.01 (−0.09–0.06)	0.04	−0.01	−0.19	0.85
Perceived Severity	0.04 (−0.06–0.13)	0.05	0.03	0.79	0.43	0.12 (0.02–0.20)	0.05	0.09	2.46	0.01	0.12 (0.03–0.19)	0.04	0.10	3.01	0.00
Response Efficacy	0.44 (0.32–0.56)	0.06	0.22	6.93	0.00	0.48 (0.34–0.61)	0.07	0.22	7.07	0.00	0.47 (0.36–0.58)	0.06	0.25	8.20	0.00
Adjusted R^2^	0.06					0.08					0.10				
